# An Importin Code in neuronal transport from synapse-to-nucleus?

**DOI:** 10.3389/fnmol.2015.00033

**Published:** 2015-07-21

**Authors:** Michael B. Lever, Anna Karpova, Michael R. Kreutz

**Affiliations:** RG Neuroplasticity, Leibniz Institute for NeurobiologyMagdeburg, Germany

**Keywords:** importins, synapse, nucleus, long-distance transport, gene expression

## Introduction

The principle cells of the brain- neurons- express more genes than any other cell type. As many rudimentary processes of the brain, such as long-lasting changes in synaptic efficacy, involve alterations to the expression of genes, the characterisation of proteins and processes that influence transcription is a high priority in neuroscience. An emerging system capable of transducing signals that likely influence transcription is synapse-to-nucleus macromolecular protein shuttling, in which synaptic proteins are relayed to the nucleus in a complex with importins (also referred to by their gene name: Karyopherins/KPNs) and molecular motors such as dynein (Jordan and Kreutz, 2009, Figure 1C). Despite a central position in a neuronal process capable of altering transcription, there is a dearth of research into importin function in neurites. In this opinion paper we summarize neuronal importin understanding to date, present novel ideas relating to their neuronal function and highlight the reductionist nature of the classical description of nuclear import with the specific example that complex composition is likely much more intricate than is being considered.

**Figure 1:**
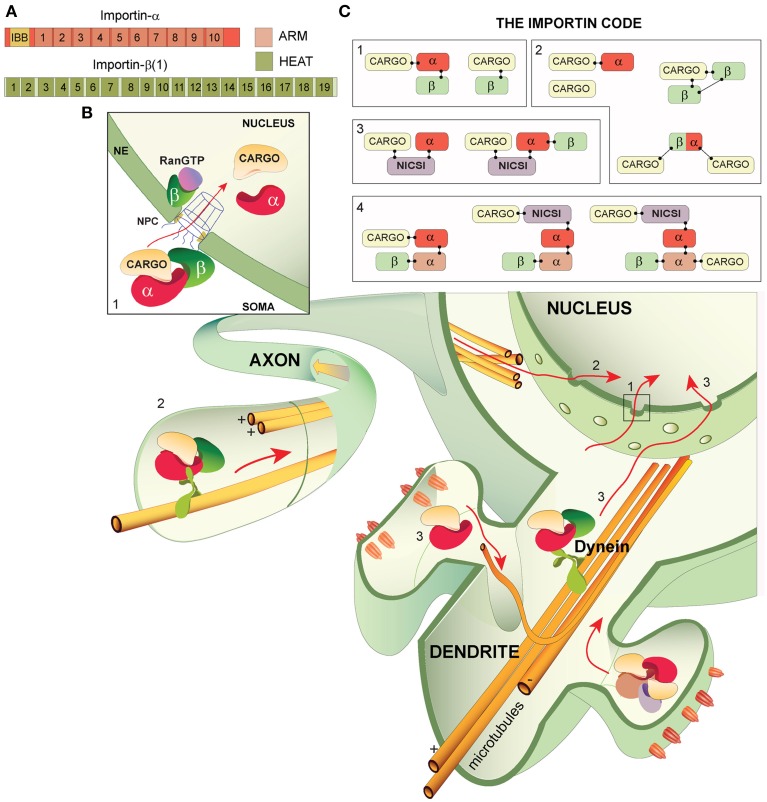
**Importin structures, canonical and non-caconocal nuclear import and The Importin Code. (A)** The general domain structure of importin-α and importin-β (1) importin-α is composed of an N-terminus importin-β binding domain (IBB) followed by 10 armadillo (ARM) repeats- cargo proteins typically bind at either ARMs 2–4 (monopartite binding at the major site) or at ARMs 2–4 and ARMs 7–8 (bipartite binding at the major and minor sites, respectively). Importin-β isoforms are comprised of 19-20 HEAT repeats. **(B)** Nuclear import in neurons from the soma, axons and dendrites/synapses: all of which may incorporate The Importin Code and converge in the nucleus where transcriptional alterations can be induced. (1) The classical description of nuclear import. The trimeric complex (importin-α, importin-β and cargo) translocates from the soma, across the nuclear pore complex (NPC) at the nuclear envelope (NE) and into the nucleus where Ran-GTP binds importin-β, liberating components of the complex. Importin-β likely transports back to the cytoplasm, whereas the cargo and importin-α may perform nuclear functions. (2) Nuclear import from axons. A complex composed of importin-α, importin-β and cargo (trimeric complex shown) is attached to microtubules via the molecular motor dynein, which drives the complex to the nucleus. (3) Synapse-to-nucleus transport. Importin-α and cargo proteins likely assemble in the synapse and are transported to the nucleus along microtubules by the molecular motor dynein. Importin-β is incorporated into the complex prior to NPC passage. **(C)** The Importin Code. (1) Classically described importin codes: the trimeric complex and importin-β alone with a cargo, (2) importin codes that are frequently attached as caveats to the classically described complex compositions. Importin-α can mediate cargo transport alone, 2 importin-β (s) can mediate transport, cargos can execute their own nuclear import independently of importins and importins may carry 2 cargos. (3) Possible importin codes incorporating the newly proposed non-importin family member cargo-specific importins (NICSIs). (4) Highly speculative importin codes based on the possibility of NICSI involvement in nuclear import and on potential importin-α dimers.

## The classical function of importins

Whilst proteins smaller than 40 kDa are free to diffuse in and out of the nucleus via the nuclear pore complex (NPC), proteins larger than this require importins. Two related families of importins have been described: Importin-α and importin-β. In humans, there are 7 importin-α isoforms (1–7) and 19 importin-β isoforms (Yasuhara et al., 2009; Xu et al., 2010; Zienkiewicz et al., 2013, Figure 1A). Importin-α subtypes comprise of an importin-β binding (IBB) domain followed by 10 armadillo (ARM) repeats that organize into 3 alpha helices (Conti et al., 1998; Kelley et al., 2010). Importin-β subtypes are composed of 19–20 HEAT repeats that arrange into a super helicoidal molecule (Xu et al., 2010). The classical pathway best describes importin understanding in nuclear import (Figure 1B1). This starts with the formation of a trimeric complex outside of the nucleus consisting of a cargo protein bound to an importin-α with its nuclear localisation signal (NLS) and the importin-α isoform bound to an importin-β with its N-terminal IBB domain (Goldfarb et al., 2004). Once formed in the cytoplasm, the trimeric complex translocates to the nucleus, either passively via diffusion or actively with the retrograde molecular motor dynein (Goldfarb et al., 2004). At the nuclear envelope, importin-β mediates the passage of the NPC, probably due to mediation with NPC-proteins FG-nucleoporins (Lott and Cingolani, 2011) and following entry into the nucleus the 100-fold higher concentration of Ran-GTP binds to importin-β, liberating the cargo for the second leg of their nuclear mission and the importins for translocation back to the cytoplasm (Conti and Izaurralde, 2001; Goldfarb et al., 2004; Stewart, 2007; Mason and Goldfarb, 2009; Ch'ng and Martin, 2011, Figure 1C). There are several exceptions to the classical description (to be discussed) but the involvement of importins in the nuclear import of cargo proteins is clear. It is also known that each importin isoform has a unique catalog of interacting partners and distinct subcellular expression (Nadler et al., 1997; Kelley et al., 2010; Schaller et al., 2014). As a result of these findings, a parsimonious hypothesis emerged: Importins function as adaptor proteins, linking distinct cargo proteins to nuclear import complexes. Therefore, by changing the expression of importins contributing to nuclear import—which is induced by activity—the nuclear proteome is shuffled, with likely alterations in transcriptional output and cellular phenotypes. As long-term forms of synaptic plasticity require such alterations in transcriptional output from the nucleus, the possibility that importins are central in this neuronal process is obvious (Thompson et al., 2004; West and Greenberg, 2011).

## Importins in synapse-to-nucleus transport

Importins exhibit a widespread distribution in dendrites, axons and synapses (Thompson et al., 2004; Jeffrey et al., 2009; Higashi-Kovtun et al., 2010). Evidence for importins having unique interacting partners in neurons comes from the finding that knockdown of importin-α1 results in improper nuclear accumulation of PER in drosophila neurons, whereas importin-α2 or importin-α3 knockdown did not have the same effect (Jang et al., 2015). Crucially, interactions and subcellular localisations of importins are altered by neuronal activity (Hanz et al., 2003; Thompson et al., 2004; Perlson et al., 2005; Dieterich et al., 2008; Jeffrey et al., 2009). This was first shown with the finding that importin-α and importin-β isoforms interact with the retrograde molecular motor dynein in the axoplasm of rats and that nerve injury induces increased association of importin-β 1 with dynein, presumably to rapidly convey the new neuronal information to the nucleus using cargo proteins such as ERK1-2 (Hanz et al., 2003; Perlson et al., 2005). This axon-to-nucleus communication of the new neuronal environment is made more efficient by the local translation of importin-β (Hanz et al., 2003). At a similar time, it emerged that importins redistribute from synaptic sites to the nucleus following the activation of synaptic receptors and that this redistribution was required for the persistence of long-term forms of synaptic plasticity (Thompson et al., 2004). Spatial and functional aspects of neuronal importins were refined with the finding that importin-α1 redistributes to the nucleus with synapse-to-nucleus messenger protein Jacob following synaptic activity (Dieterich et al., 2008) and that importin-α5 (named importin-α1 in the paper due to use of previous nomenclature) is liberated from basal associations with NMDARs following activation of these receptors, allowing nuclear accumulation (Jeffrey et al., 2009). These seminal studies give rise to the hypothesis that importins function as adaptor proteins in the mediation of cargo-specific activity-dependent synapse-to-nucleus transport. There are several caveats concerned with this description. Rather than just governing nuclear localization, neuronal importins control protein expression across several subcellular localisations: Importin-β 11 retains pMAD in the synapse and importin-α1 (importin-α2 in the paper due to unique terminology use) keeps Oct6/Brn2 in the cytoplasm (Higashi-Kovtun et al., 2010; Yasuhara et al., 2013). A strong candidate for how importins achieve this is that they maintain the current state of post-translational modifications on proteins. This is supported by the finding that enhanced neuronal cell death in importin-α5 knockout mice is rescued by the viral transfection of constitutively active STAT3 (pSTAT3), implying that importin-α5 may protect STAT3 from dephosphorylation (Ben-Yaakov et al., 2012). Emerging non-canonical functions vehemently underscore the inability of the conventional “adaptor protein” description to sufficiently depict importin functions. So, how reliably does the classical description represent importin function in nuclear import?

## Limitations of the classical pathway: Proposition of the importin code

The classical importin pathway relies on the trimeric complex (importin-β-importin-α-Cargo, see Figure 1C1), yet complex composition is known to be much more flexible. Nuclear import of cargo can be facilitated by a single importin-α (Kotera et al., 2005), single or multiple importin-β (s) (there are 11 known importin-β heterodimers) (Jäkel et al., 1999; Harel and Forbes, 2004; Xu et al., 2010) or a mechanism independent of any importin (Fagotto et al., 1998) and it remains possible that importins can carry 2 cargos at a time (Hodges et al., 2005) (Figure 1C2). As complex composition is acknowledged to be variable, what other combinations might exist? In their unraveling of the nuclear import complex involved in circadian regulation (importin-α1-TIM1-PER1) (Jang et al., 2015) find that TIM1 is evolutionarily homologous with importins and propose that it is a PER-specific importin. Likewise, Marcora and Kennedy (2010) predict that, due to similar primary and deduced secondary structure of Huntingtin (Htt) with importin-β 1, Htt may be an NFκB-specific importin in a complex composed of importin-α2, Htt and NFκB. The proposition of non-importin-family-member cargo-specific importins (NICSI's) is novel and warrants validation but it isn't a giant leap, rather, it is an extension of the existing concept of cargo-specificity in nuclear import (Figure 1C3). Because of homology to importin-β in both of the above examples, the authors predict that the NICSI replaces the importin-β in the transport complex. However, Jang et al. (2015) found that removal of the IBB domain of importin-α1 did not prevent binding of importin-α1 to TIM1, suggesting that NICSIs act as cargos to importin-α(s) and that transport complexes could feasibly be composed of importin-α, NICSI and cargo with or without an importin-β. Based on sequence similarities of over 20% identical to importin-β 1 (importin-β family members typically have between 15 and 20% homology) there are at least 5 more easily-identifiable NICSIs (MIP, BRAT1, TNPO2, STK36, SPAG6) (elucidated via BLAST search on NCBI database). As TIM1 and Htt do not meet this criterion, we expect that there are more yet-to-be-discovered NICSIs based on evolutionary homology and primary/secondary/tertiary structures. We would expect that NICSIs have unique interacting partners, in the same manner as importin-α isoforms. Other potential transport complex combinations include those expressing interactions between importin-α isoforms due to the finding that importin-α1 interacts with importin-α3 and importin-α7, and importin-α4 interacts with importin-α7 in a non-neuronal model (Kristensen et al., 2012). Assuming that some importin-α isoforms can combine with each other in neurons and that there are a number of NICSIs that are components of synapse-to-nucleus complexes, the number of potential importin-β, importin-α, importin-α and NICSI combinations becomes huge (in the order of thousands). We do not expect that all of the thousands of potential importin combinations exist but we suggest that there is a much greater number of importin combinations than is currently being portrayed by the classical description (Figure 1C4). Our prediction is that importins are involved in a highly regulatory system in cells with the expression of The Importin Code: Unique importin combinations that provide tight control over synaptic protein selection for nuclear import and govern cellular phenotypes. Just as a barcode can read and decode a specific item into the readout of a price, if we could catalog and read importin codes, we could decode synaptic proteins for nuclear import into the readout of a cellular phenotype. Very little is known about the dynamics of proteome expression in the synapse but it is estimated that there are ~2700 synaptic proteins and that ~10% possess bona fide NLSs (Jordan et al., 2004, 2007; Pielot et al., 2012). As the possession of a NLS infers ability to be translocated to the nucleus, the scope for regulating which synaptic proteins are to be transported to the nucleus is very high. Neurons thus require a highly specific system capable of selecting potentially between more than 200 synaptic proteins for activity-dependent nucleocytplasmic shuttling in accordance with the current environment. With a possibility to generate this number of combinations, The Importin Code has the capacity to be such a governing system.

## Conclusions

Whilst the hypothesis that importins function as adaptor proteins in the mediation of cargo-specific synapse-to-nucleus transport for key brain processes such as long-term forms of synaptic plasticity is informative and useful, it has several important caveats: Non-canonical importin functions are emerging and the role of importins in transport cargo specificity is likely being underestimated. The Importin Code best depicts the grand cargo specificity that importins provide to tightly regulate synaptic protein selection for nuclear import that ultimately governs cellular phenotypes.

### Conflict of interest statement

The authors declare that the research was conducted in the absence of any commercial or financial relationships that could be construed as a potential conflict of interest.
